# Lipophilic analogues of D-cysteine prevent and reverse physical dependence to fentanyl in male rats

**DOI:** 10.3389/fphar.2023.1336440

**Published:** 2024-04-05

**Authors:** James N. Bates, Paulina M. Getsy, Gregory A. Coffee, Santhosh M. Baby, Peter M. MacFarlane, Yee-Hsee Hsieh, Zackery T. Knauss, Jason A. Bubier, Devin Mueller, Stephen J. Lewis

**Affiliations:** ^1^ Department of Anesthesiology, University of Iowa Hospitals and Clinics, Iowa City, IA, United States; ^2^ Department of Pediatrics, Case Western Reserve University, Cleveland, OH, United States; ^3^ Section of Biology, Galleon Pharmaceuticals, Inc., Horsham, PA, United States; ^4^ Division of Pulmonary, Critical Care and Sleep Medicine, Case Western Reserve University, Cleveland, OH, United States; ^5^ Department of Biological Sciences, Kent State University, Kent, OH, United States; ^6^ The Jackson Laboratory, Bar Harbor, ME, United States; ^7^ Department of Pharmacology, Case Western Reserve University, Cleveland, OH, United States; ^8^ Functional Electrical Stimulation Center, Case Western Reserve University, Cleveland, OH, United States

**Keywords:** fentanyl, physical dependence, D-cysteine ethyl ester, D-cysteine ethyl amide, naloxone, withdrawal phenomena, rat

## Abstract

We examined whether co-injections of the cell-permeant D-cysteine analogues, D-cysteine ethyl ester (D-CYSee) and D-cysteine ethyl amide (D-CYSea), prevent acquisition of physical dependence induced by twice-daily injections of fentanyl, and reverse acquired dependence to these injections in freely-moving male Sprague Dawley rats. Injection of the opioid receptor antagonist, naloxone HCl (NLX, 1.5 mg/kg, IV), elicited a series of withdrawal phenomena that included cardiorespiratory and behavioral responses, and falls in body weight and body temperature, in rats that received 5 or 10 injections of fentanyl (125 μg/kg, IV), and the same number of vehicle co-injections. Regarding the development of physical dependence, the NLX-precipitated withdrawal phenomena were markedly reduced in fentanyl-injected rats that had received co-injections of D-CYSee (250 μmol/kg, IV) or D-CYSea (100 μmol/kg, IV), but not D-cysteine (250 μmol/kg, IV). Regarding reversal of established dependence to fentanyl, the NLX-precipitated withdrawal phenomena in rats that had received 10 injections of fentanyl (125 μg/kg, IV) was markedly reduced in rats that received co-injections of D-CYSee (250 μmol/kg, IV) or D-CYSea (100 μmol/kg, IV), but not D-cysteine (250 μmol/kg, IV), starting with injection 6 of fentanyl. This study provides evidence that co-injections of D-CYSee and D-CYSea prevent the acquisition of physical dependence, and reverse acquired dependence to fentanyl in male rats. The lack of effect of D-cysteine suggests that the enhanced cell-penetrability of D-CYSee and D-CYSea into cells, particularly within the brain, is key to their ability to interact with intracellular signaling events involved in acquisition to physical dependence to fentanyl.

## Introduction

The continued use of opioids is associated with development of physical dependence, as evidenced by behavioral and cardiorespiratory phenomena, which occur upon caesing opioid use (i.e., spontaneous withdrawal) or administration of an opioid receptor antagonist (i.e., precipitated withdrawal) (Garcia et al., 2014; Volkow et al., 2018; Mercadante et al., 2019). At present, there are no therapeutics that prevent acquisition of physical dependence to opioids. Although none have proven to be clinically effective, some agents have shown some ability to reverse physical dependence to opioids, including the β_2_-AR antagonist, butoxamine (Liang et al., 2007), adrenomedullin receptor antagonists (Wang et al., 2011), and allosteric modulators of the AMPA (α-amino-3-hydroxy-5-methyl-4-isoxazolepropionic acid) glutamate receptors (Hu et al., 2018). Other agents include L-histidine and histamine receptor agonists (Wong and Roberts, 1976), the dopamine D2 receptor antagonist, haloperidol (Yang et al., 2011), ATP-dependent K^+^-channel modulators (Singh et al., 2015), inhibitors of Ca^2+^-calmodulin-dependent protein kinase II (Wang et al., 2003; Tang et al., 2006), the serotonin-reuptake inhibitor, fluoxetine (Singh et al., 2003), nitric oxide synthase inhibitors (Naidu et al., 2003; Singh et al., 2003), and the antioxidants melatonin (Raghavendra and Kulkarni, 1999; 2000) and quercetin (Singh et al., 2002; Naidu et al., 2003). The diversity of these therapeutics speaks to our lack of understanding as to the mechanisms by which physical dependence to opioids occurs.

**FIGURE 1:**
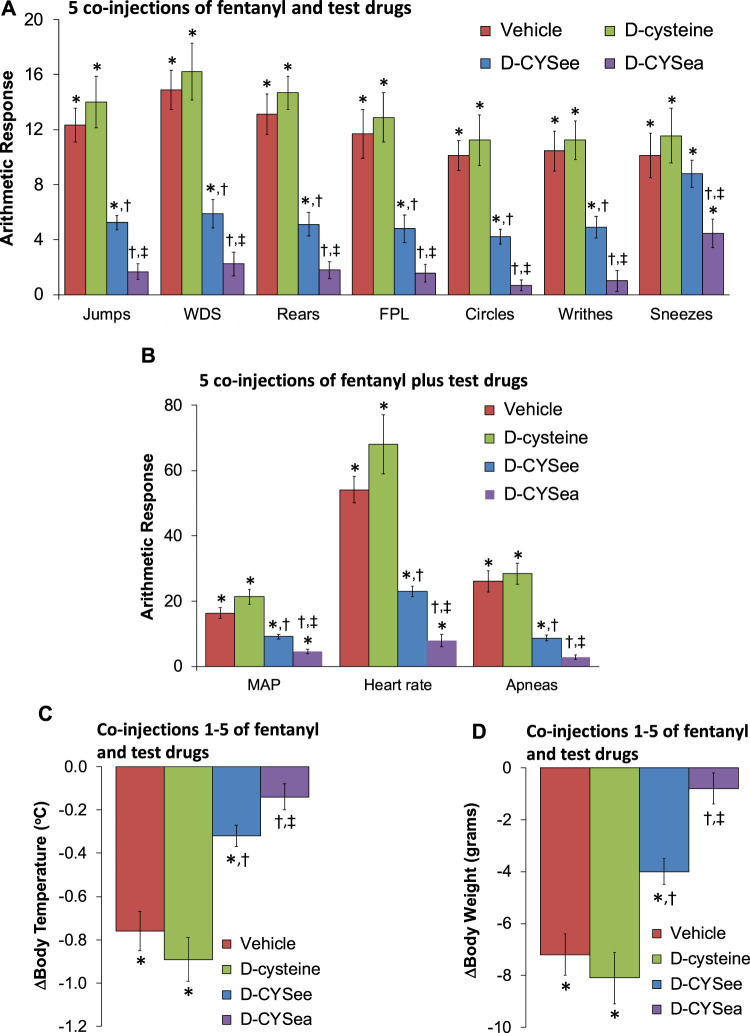
Responses elicited by the injection of naloxone HCl (1.5 mg/kg, IV) in rats that had received 5 injections of fentanyl (125 μg/kg, IV) plus 5 co-injections of vehicle, D-cysteine (250 μmol/kg, IV), D-cysteine ethyl ester (D-CYSee, 250 μmol/kg, IV), or D-cysteine ethyl amide (D-CYSea, 100 μmol/kg, IV). **(A)** Behavioral responses—jumps, wet-dog shakes (WDS), rears, fore-paw licking (FPL), circles, writhes, and sneezes. **(B)** Cardiorespiratory responses, mean arterial blood pressure (MAP), heart rate, and apneas. **(C)** Body temperature. **(D)** Body weight. Data are shown as mean ± SEM. There were 9 rats in each group. **ANOVA statistics: (A)** Jumps (F_3,32_ = 28.0, *p* < 0.0001); WDS (F_3,32_ = 22.2, *p* < 0.0001); Rears (F_3,32_ = 47.3, *p* < 0.0001); FPL (F_3,32_ = 24.3, *p* < 0.0001); Circles (F_3,32_ = 20.5, *p* = 0.0009); Writhes (F_3,32_ = 20.0, *p* < 0.0001); Sneezes (F_3,32_ = 4.34, *p* = 0.011). **(B)** MAP (F_3,32_ = 31.7, *p* < 0.0001); Heart rate (F_3,32_ = 38.2, *p* < 0.0001); Apneas (F_3,32_ = 32.7, *p* < 0.0001). **(C)** Body temperature (F_3,32_ = 23.5, *p* < 0.0001). **(D)** Body weight (F_3,32_ = 18.1, *p* < 0.0001).*p < 0.05, significant response from Pre-values. ^†^ *p* < 0.05, D-CYSea or D-CYSee versus vehicle. ^‡^ *p* < 0.05, D-CYSea versus D-CYSee.

Trivedi et al. (2014) provided evidence that morphine-induced redox-dependent changes in DNA methylation and retrotransposon transcription status via inhibition of excitatory amino acid transporter type 3 (EAAT3)-mediated uptake of cysteine into brain neurons, may be causal to the development of physical dependence to morphine. Possible mechanisms suggested from the studies of Trivedi et al. (2014) and others (Lin et al., 2001; Ikemoto et al., 2002; Mao et al., 2002; Xu et al., 2003; Xu et al., 2006; Christie, 2008; Yang et al., 2008; Wang et al., 2009; Daijo et al., 2011; Gutowicz et al., 2011; Liu et al., 2011; Maze and Nestler, 2011; Lim et al., 2012; Sun et al., 2012; Browne et al., 2020) involve (a) morphine-induced attenuation of L-cysteine uptake into neurons by G-protein-mediated decrease in EAAT3 function, (b) decreases in intracellular L-cysteine and L-glutathione, (c) decreases in methylation index (S-adenosyl-methionine/S-adenosyl-homocysteine), (d) reduced methylation of global CpG (regions of DNA in which a cytosine nucleotide is followed by a guanine nucleotide in linear base sequence along the 5′ → 3′ direction), (e) decreased CpG methylation of long interspersed nuclear element–1 (LINE-1) retrotransposon regulatory regions, and (f) activation of transcription of previously silenced LINE-1 gene (see Figures 1 and 7 of Trivedi et al., 2014).

On the basis of these findings we examined whether co-administration of the cell-penetrant L-thiol ester, L-cysteine ethyl ester (L-CYSee), would reduce physical dependence to morphine in male Sprague Dawley rats and overcome established dependence to the opioid (Bates et al., 2023). With respect to preventing the acquisition of dependence, we found that the injection of the opioid-receptor antagonist, naloxone HCl (NLX; 1.5 mg/kg, IP), elicited pronounced withdrawal phenomena in rats which received a subcutaneous depot of morphine (150 mg/kg) for 36 h and a continuous infusion of saline (20 μL/h, IV) via osmotic minipumps for this 36 h period. Withdrawal phenomena observed were wet-dog shakes (WDS), jumping, rearing, fore-paw licking (FPL), 360^o^ circling, writhing, apneas, cardiovascular (e.g., pressor, tachycardic) responses, hypothermia, and body weight loss. Remarkably, NLX elicited fewer phenomena in rats that received an infusion of L-CYSee (20.8 μmol/kg/h, IV) for 36 h. With respect to reversing acquired dependence, we first established that NLX precipitated marked withdrawal syndrome in rats that had received subcutaneous depots of morphine (150 mg/kg) for 48 h and a co-infusion of vehicle. Again, the NLX-precipitated withdrawal phenomena were reduced in morphine-treated (150 mg/kg for 48 h) rats that began receiving an infusion of L-CYSee (20.8 μmol/kg/h, IV) at 36 h. Of equal importance was that infusion of L-cysteine or L-serine ethyl ester, both at 20.8 μmol/kg/h, IV, did not mimic the effects of L-CYSee. As such, L-CYSee may attenuate the development of physical dependence to morphine in rats, and reverse dependence acquired before administration of L-CYSee, most likely by intracellular actions in the brain. The lack of effect of L-serine ethyl ester, which contains an oxygen atom instead of a sulfur atom, implicates thiol biochemistry in the efficacy of L-CYSee. These characteristics of L-CYSee provides supporting evidence to the ability of this (Lewis et al., 2022), and other L-thiolesters (Jenkins et al., 2021; Getsy et al., 2022a), to prevent and/or reverse the actions of morphine and fentanyl on ventilatory parameters, and arterial blood-gas chemistry in rats without compromising opioid-induced analgesia or sedation. In pursuing mechanisms of action of the L-thiol esters, we found that D-thiol esters, such as D-cysteine ethyl ester (D-CYSee), D-cystine diethyl ester (D-CYSdiee), and D-cysteine dimethyl ester (D-CYSdime), also reversed the adverse effects of morphine on breathing without compromising analgesia (Gaston et al., 2021; Getsy et al., 2022b; Getsy et al., 2022c).

We recently reported that the co-administration of D-CYSee with fentanyl prevents the development of fentanyl-induced conditioned place preference in male and female rats (Knauss et al., 2023), thus suggesting D-CYSee likely reduces the rewarding properties of fentanyl and therefore reduces its addictive potential. The question that arose from these studies was whether D-CYSee can prevent acquisition of physical dependence to fentanyl and/or reverse established dependence to this powerful synthetic opioid. Another important question was whether D-cysteine ethyl amide (D-CYSea) has greater efficacy than D-CYSee based on its expected enhanced resistance to plasma carboxylesterases that potentially convert D-CYSee to D-cysteine (Butterworth et al., 1993; Nishida et al., 1996). The present study demonstrates that co-injections of D-CYSee and D-CYSea prevent and reverse the acquisition of fentanyl physical dependence in male rats, and that D-CYSea appears to be more efficacious compared to D-CYSee. As such, these D-cysteine analogues may represent a novel class of therapeutics that ameliorate the development of physical dependence to opioids in humans.

## Materials and methods

### Permissions, rats, and surgical procedures

All studies were done according to the NIH Guide for Care and Use of Laboratory Animals (NIH Publication No. 80–23) revised in 1996, and in compliance with ARRIVE (Animal Research: Reporting of *In Vivo* Experiments) guidelines (http://www.nc3rs.org.uk/ page. asp? id = 1357). All protocols involving the use of rats were approved by the Animal Care and Use Committees of the University of Virginia and *Galleon Pharmaceuticals*. A total of 578 adult male Sprague Dawley rats (*n* = 9 rats in each study group) purchased from *Harlan Industries* (Madison, WI, United States) were used in these studies (see Supplementary Table S1 for information including body weights at the time of starting each protocol). The rats were given 5 days to recover from transportation in our Animal Resource Center (12 h light/12 h dark cycle, lights off at 6 p.m./lights on at 6 a.m.; room humidity of 49% ± 3%; room temperature of 21.5°C ± 0.3°C; standard corn cob bedding from *Gateway Labsupply*, St. Louis, MO, United States) before undergoing surgery. Rats were also given 5 days to recover from surgery in our Animal Resource Center before use in experiments (conditions as above except for the bedding, which was ALPHAdri bedding from *Lab Supply*, Durham, NC). The rats had free access to water at all times. The rats had free access to food at all times, except for the 90 min period immediately following completion of the surgery. Fentanyl citrate powder was obtained from *Sigma-Aldrich* (St. Louis, MO, United States). D-CYSee and D-CYSea were obtained from *ChemImpex* (Wood Dale, Illinois, United States) respectively, and *Olon RIcerca* (Concord, Ohio, United States) respectively, and divided into 100 mg amounts under N_2_ gas and stored at 4°C. Solutions of D-CYSee and D-CYSea (dissolved in saline and brought to pH 6.8 with 0.1 M NaOH at room temperature) were prepared immediately before use. Naloxone HCl (*Sigma-Aldrich*, St. Louis, MO, United States) was dissolved in normal saline. All arterial and venous catheters were flushed with 0.3 mL of phosphate-buffered saline (0.1 M, pH 7.4) 3–4 h before starting the study. All studies were done in a room with relative humidity of 49% ± 2% and temperature of 21.4°C ± 0.2°C. Note that each rat was used in only one of the study protocols described below and was not used in any other study. Finally, all independent observers and experimenters were blinded to the specific treatments that the rats had undergone in every study protocol described below.

### D-CYSee or D-CYSea prevention of the development of physical dependence to fentanyl

#### Behavioral studies

Three groups of rats were implanted with a catheter composed of PE-10 tubing connected to PE-50 tubing (*Intramedic; Becton & Dickinson, Franklin Drive, NJ, United States*) into the jugular vein under 2%–3% isoflurane anesthesia delivered in 60% O_2_, to allow injections of test agents (May et al., 2013a; May et al., 2013b; Henderson et al., 2013; Henderson et al., 2014). The jugular vein IV lines in this and all protocols described hereafter were kept patent during the recovery period by injecting a bolus volume of saline (300 μL) once daily. **Study 1–5 co-injections:** Rats received co-injections of vehicle (100 μL/100 g body weight, IV) + fentanyl (125 μg/kg, IV), D-cysteine (250 μmol/kg, IV) + fentanyl (125 μg/kg, IV), D-CYSee (250 μmol/kg, IV) + fentanyl (125 μg/kg, IV), or D-CYSea (100 μmol/kg, IV) + fentanyl (125 μg/kg, IV) given 90 s apart (fentanyl given second in all instances) at 8 a.m. and 8 p.m. on days 1 and 2, and at 8 a.m. on day 3. **Study 1–10 co-injections:** Rats received co-injections of vehicle (100 μL/100 g body weight, IV) + fentanyl (125 μg/kg, IV), D-cysteine (250 μmol/kg, IV) + fentanyl (125 μg/kg, IV), D-CYSee (250 μmol/kg) + fentanyl (125 μg/kg, IV) or D-CYSea (100 μmol/kg, IV) + fentanyl (125 μg/kg, IV) given 90 s apart at 8 a.m. and 8 p.m. on days 1–4. The rats were given injection 9 at 8 a.m. on day 5 and injection 10 at 2 p.m. to allow for NLX challenges to be given. Ninety min after the 5th or 10th set of co-injections, rats were placed in individual opaque plastic boxes and after 30 min, they were injected with NLX (1.5 mg/kg, IV) and behavioral phenomena were scored for 45 min by 3 scorers. Scored behavioral phenomena were: Jumping behavior—all 4 paws off the ground—jumps; Wet dog shakes—whole body shakes as if to shed water from fur; Rearing behavior—rearing on hind legs—rears; Episodes of fore-paw licking—FPL; Circling behavior—Complete 360^o^ rotation; Writhes—full body contortion; Episodes of sneezing—abrupt expulsion of air that often disturbed the fine bedding material-sneezes. Other groups of rats (*n* = 9 rats per group, see Supplementary Table S1) underwent the same protocols as above, except that the rats received an injection of vehicle instead of fentanyl (i.e., vehicle + vehicle injections, vehicle + D-CYSee injections, or vehicle + D-CYSea injections). These rats also received an injection of NLX (1.5 mg/kg, IV) given 90 min after the last set of injections.

#### Plethysmography ventilatory studies

One hour before co-injections 5 or 10 were to be given (as described above), rats were placed into individual whole body plethysmography chambers to record ventilatory parameters (Jenkins et al., 2021; Lewis et al., 2022; Getsy et al., 2022a; Getsy et al., 2022b; Getsy et al., 2022c). The free end of the venous catheter was attached to a swivel assembly in the lid of the chamber and after 60 min of acclimatization, the rats received co-injections 5 or 10, and after 90 min they received an injection of NLX (1.5 mg/kg, IV). The number of apneas of greater than 1.5 s in duration were determined by internal *FinePointe* software (DSI, Harvard Bioscience, Inc., St. Paul, MN) (Getsy et al., 2022a; Getsy et al., 2022b; Getsy et al., 2022c; Getsy et al., 2022d; Getsy et al., 2022e; Lewis et al., 2022).

#### Cardiovascular studies

Rats were implanted with a jugular vein catheter to inject drugs, and a femoral artery catheter to record mean arterial blood pressure (MAP) and heart rate (Hashmi-Hill et al., 2007; Kanbar et al., 2010; Davisson et al., 2014; Brognara et al., 2016). The rats were given 4 days to recover. The jugular vein IV lines were kept patent during the recovery period by injecting a bolus volume of saline (300 μL) once daily. The patency of arterial lines was maintained by connecting the line to an infusion pump (*Standard Infuse-Withdraw Pump 11 Pico Plus Elite Programmable Syringe Pump; Harvard Apparatus, MA, United States*) delivering saline at 20 μL/h (Getsy et al., 2022d; Getsy et al., 2022e). Sixty min before co-injections 5 or 10 were to be administered, the rats were put into individual opaque plastic boxes in order to administer drugs, and to actively record pulsatile arterial blood pressure (MAP) and heart rate. After 60 min acclimatization, the rats received co-injections 5 or 10, and then, after 90 min, they received NLX (1.5 mg/kg, IV) and cardiovascular parameters recorded for a further 90 min.

#### Body temperature and body weight studies

Rats were put into individual opaque plastic boxes 1 h before co-injections 5 or 10 of the fentanyl + drug combinations were to be given. A thermistor probe attached to a telethermometer (*Yellow Springs Instruments*) to record body temperature was inserted 5–6 cm into the rectum and taped to the tail (Lewis et al., 1986). Body weights and body temperatures were recorded every 15 min during the acclimatization period to establish baseline values, and at 15 min intervals during the injection protocols. After 60 min of acclimatization, rats received co-injections 5 or 10, and after 90 min an injection of NLX (1.5 mg/kg, IV), and then body weights and body temperatures were recorded for another 90 min.

### Protocols to determine the abilities of D-CYSee or D-CYSea to reverse fentanyl dependence

#### Behavioral studies

Rats received 5 injections of fentanyl (125 μg/kg, IV) at 8 a.m. and 8 p.m. as described above. The rats then received co-injections 6–10 of fentanyl (125 μg/kg, IV) + vehicle, D-cysteine (250 μmol/kg, IV) + fentanyl (125 μg/kg, IV), D-CYSee (250 μmol/kg, IV) + fentanyl (125 μg/kg, IV) or D-CYSea (100 μmol/kg, IV) + fentanyl (125 μg/kg, IV) given 90 s apart. Co-injections 6 were given at 8 p.m., co-injections 7 at 8 a.m., co-injections 8 at 8 p.m., co-injections 9 at 8 a.m., and co-injection 10 was given at 2 p.m. to allow for the experiments to be performed. Immediately after co-injections 10 were given, the rats were placed in individual opaque plastic boxes, and after a 90 min acclimatization period, the rats received an injection of NLX (1.5 mg/kg, IV), and behavioral phenomena (as detailed above) were scored for 45 min by at least 3 independent scorers.

#### Plethysmography ventilatory studies

One hour before co-injections 10 were to be given, rats were put into individual whole body plethysmography chambers to record ventilatory parameters. The free end of the externalized venous catheter was connected to the swivel assembly and, after 60 min acclimatization, the rats received the 10th set of co-injections and after 90 min they received NLX (1.5 mg/kg, IV). Ventilatory parameters and non-eupneic breathing indices were recorded (to be reported elsewhere) with the number of apneas recorded as >1.5 s in duration reported here.

#### Cardiovascular studies

One hour before the 10th set of co-injections were given, groups of rats (*n* = 9 rats per group) were put in individual opaque plastic boxes, and the free end of the exteriorized jugular vein catheter was connected to an injection line to give drugs. The free end of the arterial catheter was then connected to tubing attached to a computer-coupled pressure transducer (*Cabe Lab, Inc.*) to continuously record pulsatile arterial blood pressure to derive MAP and heart rate. After 60 min acclimatization, rats received co-injections 5 or 10, and then after 90 min, an injection of NLX (1.5 mg/kg, IV), and cardiovascular parameters were recorded continuously for a further 90 min.

#### Body temperature and body weight studies

Rats were put into individual opaque plastic boxes 1 h before the 10th set of co-injections were to be given. A thermistor probe for body temperature recordings was placed as above. Rat body weights and temperatures were recorded every 15 min during acclimatization to establish baseline values, and at 15 min intervals throughout the injection protocols. After 60 min, the rats received co-injections 10, and after 90 min, they were injected with NLX (1.5 mg/kg, IV), and body weights and body temperatures were recorded for another 90 min.

### Data analyses

**FIGURE 2:**
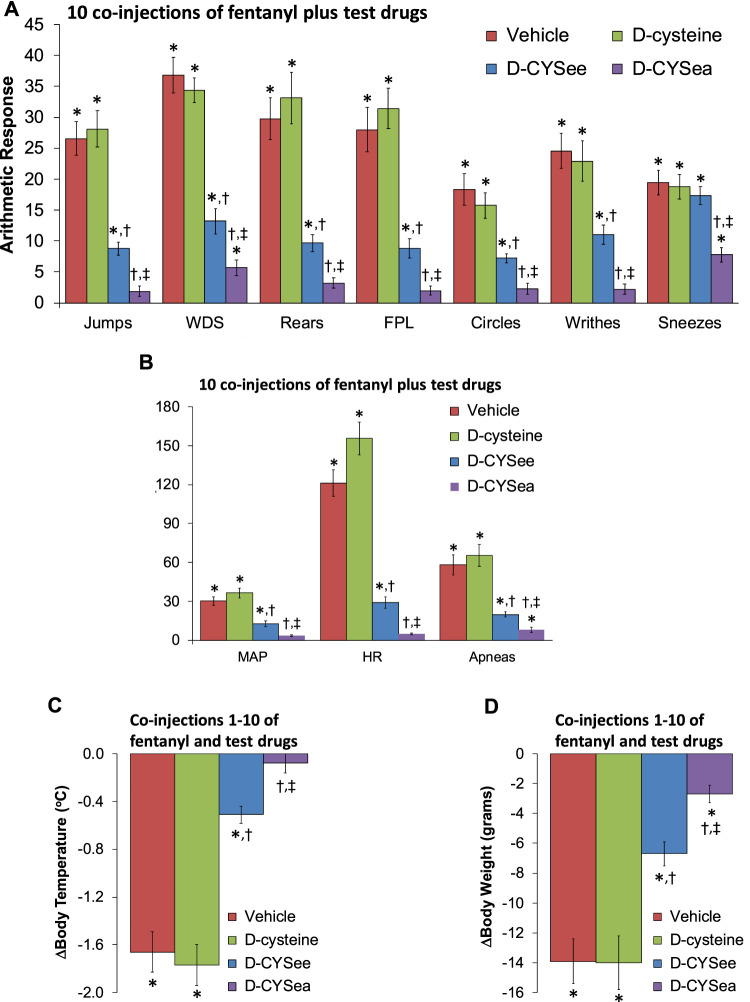
Responses elicited by the injection of naloxone HCl (1.5 mg/kg, IV) in rats that had received 10 injections of fentanyl (125 μg/kg, IV) plus 10 co-injections of vehicle, D-cysteine (250 μmol/kg, IV), D-cysteine ethyl ester (D-CYSee, 250 μmol/kg, IV), or D-cysteine ethyl amide (D-CYSea, 100 μmol/kg, IV). **(A)** Behavioral responses—jumps, wet-dog shakes (WDS), rears, fore-paw licking (FPL), circles, writhes, and sneezes. **(B)** Cardiorespiratory responses, mean arterial blood pressure (MAP), heart rate, and apneas. **(C)** Body temperature. **(D)** Body weight. Data are shown as mean ± SEM. There were 9 rats in each group. **ANOVA statistics: (A)** Jumps (F_3,32_ = 48.5, *p* < 0.0001); WDS (F_3,32_ = 38.7, *p* < 0.0001); Rears (F_3,32_ = 34.7, *p* < 0.0001); FPL (F_3,32_ = 42.8, *p* < 0.0001); Circles (F_3,32_ = 19.3, *p* = 0.0009); Writhes (F_3,32_ = 30.2, *p* < 0.0001); Sneezes (F_3,32_ = 6.65, *p* = 0.001). **(B)** MAP (F_3,32_ = 37.6, *p* < 0.0001); Heart rate (F_3,32_ = 88.4, *p* < 0.0001); Apneas (F_3,32_ = 25.8, *p* < 0.0001). **(C)** Body temperature (F_3,32_ = 48.4, *p* < 0.0001). **(D)** Body weights (F_3,32_ = 18.5, *p* < 0.0001).*p < 0.05, significant response from Pre-values. ^†^ *p* < 0.05, D-CYSea or D-CYSee versus vehicle. ^‡^ *p* < 0.05, D-CYSea versus D-CYSee.

**FIGURE 3:**
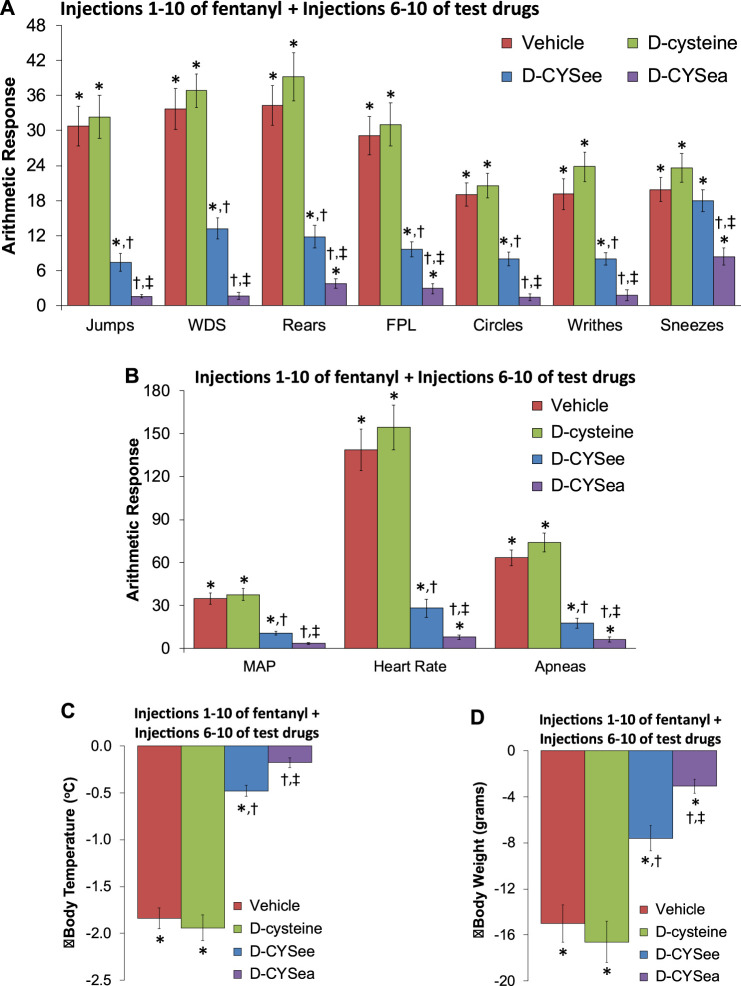
Responses elicited by the injection of naloxone HCl (1.5 mg/kg, IV) in rats that had received 10 injections of fentanyl (125 μg/kg, IV) plus 5 co-injections of vehicle, D-cysteine (250 μmol/kg, IV), D-cysteine ethyl ester (D-CYSee, 250 μmol/kg, IV), or D-cysteine ethyl amide (D-CYSea, 100 μmol/kg, IV) beginning at fentanyl injection 6. **(A)** Behavioral responses—jumps, wet-dog shakes (WDS), rears, fore-paw licking (FPL), circles, writhes, and sneezes. **(B)** Cardiorespiratory responses, mean arterial blood pressure (MAP), heart rate, and apneas. **(C)** Body temperature. **(D)** Body weight. Data are shown as mean ± SEM. There were 9 rats in each group. ANOVA statistics: **(A)** Jumps (F_2,24_ = 36.2, *p* < 0.0001); WDS (F_2,24_ = 46.8, *p* < 0.0001); Rears (F_2,24_ = 35.8, *p* < 0.0001); FPL (F_2,24_ = 29.8, *p* < 0.0001); Circles (F_2,24_ = 32.2, *p* = 0.0009); Writhes (F_2,24_ = 33.1, *p* < 0.0001); Sneezes (F_2,24_ = 8.6, P = < 0.0003). **(B)** MAP (F_2,24_ = 31.9, *p* < 0.0001); Heart rate (F_2,24_ = 45.3, *p* < 0.0001); Apneas (F_2,24_ = 52.7, *p* < 0.0001). **(C)** Body temperature (F_2,24_ = 86.6, *p* < 0.0001). **(D)** Body weight (F_2,24_ = 22.4, *p* < 0.0001). *p < 0.05, significant response from Pre-values. ^†^ *p* < 0.05, D-CYSea or D-CYSee versus vehicle. ^‡^ *p* < 0.05, D-CYSea versus D-CYSee.

All data are presented as mean ± SEM. All between-group data were analyzed by one-way ANOVA as detailed previously (Getsy et al., 2023a; Getsy et al., 2023b). The statistical analyses were performed with the aid of GraphPad Prism software (Version 9.5.1–2023; *GraphPad Software*, Inc., La Jolla, CA). The F- and *P*-statistics related to Figures 1–3 are provided in the relevant figure legends.

## Results

### NLX does not elicit withdrawal behaviors in rats that did not receive co-injections of fentanyl

The administration of NLX (1.5 mg/kg, IV) to rats that had received the same injection protocols of D-CYSee (250 μmol/kg, IV) or D-CYSea (100 μmol/kg, IV), but with co-injections of vehicle, rather than fentanyl, are presented in Supplementary Tables S2–S4. As seen in Supplementary Table S2, the injection of NLX elicited virtually no behavioral responses in rats that had received 5 or 10 co-injections of vehicle + vehicle, vehicle + D-CYSee, or vehicle + D-CYSea, or in rats that received 10 injections of vehicle plus co-injections of vehicle, D-CYSee, or D-CYSea beginning with injection 6 of vehicle. As seen in Supplementary Table S3, the body weights of the rats grew as expected during the course of the protocols, in the rats that received co-injections of vehicle + vehicle. The increases in body weights were similar in the rats that received co-injections of D-CYSee or D-CYSea. As also seen in Supplementary Table S3, there were no between-treatment group differences in body temperature at any stage of the Inj1-5 or Inj1-10 protocols. Importantly, the injection of NLX did not precipitate any changes in body weight or body temperature in any treatment group. As seen in Supplementary Table S4, the body weights of the rats grew as expected during the course of the co-Inj6-10 protocols, with no-between-group differences being found. The increases in body weights were similar in rats that received co-injections of D-CYSee or D-CYSea. Again, body temperatures remained equivalent throughout the study protocols. The administration of NLX did not elicit changes in body weight or body temperature in any of these treatment groups.

### D-CYSee and D-CYSea prevent physical dependence to fentanyl

The behaviors elicited by the injection of NLX in rats that had received 5 co-injections of fentanyl + vehicle, D-cysteine, D-CYSee, or D-CYSea are summarized in Figure 1A. The injection of NLX in fentanyl + vehicle-injected rats produced jumps, wet-dog shakes (WDS), rearing, fore-paw licking (FPL), circling, full-body writhing, and episodes of sneezing. These NLX-precipitated responses were similar in rats that had received co-injections of fentanyl + D-cysteine. Except for sneezing, these NLX-precipitated behaviors were reduced in rats that received fentanyl + D-CYSee, and much more so in rats that had received fentanyl + D-CYSea. As summarized in Figure 1B, the injection of NLX produced sustained increases in MAP and heart rate, and elicited a large increase in apneas (>1.5 s between breaths). The NLX-induced responses were reduced in rats that received co-injections of fentanyl + D-CYSee and much more so in rats that received co-injections of fentanyl + D-CYSea. As summarized in Figures 1C, D, the NLX-induced decreases in body weight and body temperature were smaller in rats that received fentanyl + D-CYSee, and markedly less in rats that received fentanyl + D-CYSea, but not in those that received D-cysteine. Behaviors elicited by NLX in rats that received 10 co-injections of fentanyl + vehicle, D-cysteine, D-CYSee, or D-CYSea are summarized in Figure 2A. Administration of NLX to rats that received fentanyl + vehicle produced qualitatively similar responses as described above in Figure 1A, except that they were more intense. NLX-precipitated withdrawal phenomena were not diminished in rats that received co-injections of D-cysteine, whereas, except for sneezing, the responses were reduced in rats that had received fentanyl + D-CYSee. Additionally, all NLX-precipitated responses (including sneezing) were markedly reduced in rats that received co-injections of D-CYSea, and these responses were less than in those that received D-CYSee. As summarized in Figure 2B, the NLX-induced increases in MAP, heart rate, and incidences of apneic events (>1.5 s between breaths) were reduced in rats that received co-injections of D-CYSee (but not D-cysteine), and more so in the rats that received D-CYSea. As summarized in Figures 2C, D, the NLX-precipitated falls in body weight and body temperature were reduced in rats that had received fentanyl + D-CYSee, and markedly diminished in rats that had received fentanyl + D-CYSea.

### D-CYSee and D-CYSea reverse established physical dependence to fentanyl

The behaviors elicited by injection of NLX in rats that had received 10 injections of fentanyl plus 5 co-injections of vehicle, D-cysteine, D-CYSee, or D-CYSea starting with fentanyl injection 6 are summarized in Figure 3A. The responses seen after injection of NLX were similar in rats that received co-injections of vehicle or D-cysteine. Moreover, except for sneezing, the NLX-precipitated behaviors were reduced in rats that received co-injections of D-CYSee. Additionally, all behaviors were markedly diminished in rats that had received co-injections of D-CYSea. As summarized in Figure 3B, the NLX-precipitated elevations in MAP, heart rate, and incidences of apneas (>1.5 s between breaths), were diminished in rats that received D-CYSee, and markedly diminished in rats that received D-CYSea. As summarized in Figures 3C, D, the NLX-induced falls in body weight and body temperature were less in rats that received fentanyl + D-CYSee, and markedly diminished in rats that received fentanyl + D-CYSea.

### Changes in variables during the progression of the protocols

Body weights and arithmetic changes in these weights at key points of the three study protocols are shown in Table 1. The rats that received the 5 or 10 co-injections of fentanyl + vehicle or fentanyl + D-cysteine, lost body weight (arithmetic change from Pre-drug values, i.e., Pre). In contrast, body weights rose in rats receiving co-injections of D-CYSee and D-CYSea. In rats that received fentanyl + vehicle, the loss in body weight after 10 injections was greater than after 5 injections. In contrast, the increases in body weight in rats that received fentanyl + D-CYSee or fentanyl + D-CYSea after the 10 co-injections were greater than after the 5 co-injections. The decreases in body weight elicited by NLX were similar in rats that received vehicle or D-cysteine, but markedly less in rats that received co-injections of D-CYSee or D-CYSea. With respect to the co-injection 6–10 study, the rats that received co-injections of fentanyl + vehicle and fentanyl + D-cysteine lost body weight, whereas those that received co-injections of fentanyl + D-CYSee or D-CYSea gained weight. The NLX-induced decreases in body weight seen in the rats that received co-injections of D-cysteine were similar to those that received co-injections of vehicle. The falls in body weight were again smaller in the rats that received co-injections of D-CYSee, and even less in rats that received D-CYSea. Actual and arithmetic changes in body temperatures at key points of co-injections 1–5 and 1–10 studies and co-injection 6–10 study are summarized in Table 2. The rats that received 5 or 10 co-injections of fentanyl + vehicle developed a hyperthermia that also occurred in rats that received fentanyl + D-cysteine, but not in rats that received fentanyl + D-CYSee or fentanyl + D-CYSea. The injection of NLX elicited a profound hypothermia in rats that received co-injections of vehicle or D-cysteine, but not in those that received D-CYSee or D-CYSea. With respect to the co-injection 6–10 study, the rats that received co-injections of fentanyl + vehicle or fentanyl + D-cysteine, showed a pronounced hyperthermia, whereas those that received D-CYSee or D-CYSea did not. A NLX-precipitated hypothermia was seen in the rats that received co-injections of vehicle or D-cysteine. This hypothermia was less in rats that received co-injections of D-CYSee, and substantially less in rats that received D-CYSea.

**TABLE 1:** Body weights at key points of each of the study protocols.

Co-injection 1–5 Study	Stage	Actual body weight (grams) and arithmetic changes in weight			
		Vehicle	D-cysteine	D-CYSee	D-CYSea
Behavioral	Pre	337 ± 2	336 ± 1	337 ± 2	337 ± 1
MAP and Heart Rate	Pre	338 ± 2	337 ± 2	338 ± 2	339 ± 2
Apnea	Pre	337 ± 2	337 ± 2	337 ± 1	339 ± 2
Body weight and body temperature	Pre	338 ± 2	338 ± 2	337 ± 1	338 ± 2
	Post-inj 5	332 ± 1	331 ± 2	342 ± 1	345 ± 2
	Post-NLX	325 ± 1	323 ± 2	340 ± 1	344 ± 2
	Post-inj 5 vs. Pre	−5.8 ± 0.5*	−6.9 ± 0.9*	+4.8 ± 0.6*^,†^	+7.7 ± 1.1*^,†,‡^
	Post-NLX vs. Post-inj 5	−6.9 ± 0.8*	−8.1 ± 1.0*	−2.2 ± 0.4*^,†^	−1.1 ± 0.3*^,†^
Co-injection 1–10 Study	Stage	Vehicle	D-cysteine	D-CYSee	D-CYSea
Behavioral	Pre	337 ± 1	338 ± 2	337 ± 2	340 ± 2
MAP and Heart Rate	Pre	337 ± 2	338 ± 2	339 ± 2	337 ± 2
Apnea	Pre	337 ± 2	336 ± 2	338 ± 2	337 ± 2
Body weight and body temperature	Pre	338 ± 2	336 ± 2	338 ± 2	337 ± 2
	Post-inj 10	329 ± 1	324 ± 2	345 ± 2	348 ± 3
	Post-NLX	315 ± 2	310 ± 3	341 ± 2	344 ± 2
	Post-inj 10 vs. Pre	−9.6 ± 1.4*	−11.8 ± 1.9*	+7.3 ± 1.3*^,†^	+10.8 ± 1.6*^,†^
	Post-NLX vs. Post-inj 10	−13.9 ± 1.5*	−14.0 ± 1.8*	−4.1 ± 0.8*^,†^	−4.0 ± 1.0*^,†^
Co-injection 6–10 Study	Stage	Vehicle	D-cysteine	D-CYSee	D-CYSea
Behavioral	Pre- prior to injection 6	329 ± 1	330 ± 2	349 ± 2	352 ± 2
MAP and Heart Rate	Pre	330 ± 2	329 ± 2	350 ± 2	354 ± 2
Apnea	Pre	330 ± 2	328 ± 2	350 ± 2	351 ± 2
Body weight and body temperature	Pre	330 ± 2	331 ± 1	349 ± 2	350 ± 2
	Post-inj 10	319 ± 1	317 ± 2	359 ± 2	362 ± 1
	Post-NLX	304 ± 2	301 ± 2	352 ± 2	359 ± 2
	Post-inj 10 vs. Pre	−10.9 ± 1.2*	−13.4 ± 1.4*	+10.8 ± 1.6*^,†^	+11.2 ± 1.8*^,†^
	Post-NLX vs. Post-inj 10	−15.0 ± 1.6*	−16.6 ± 1.8*	−7.6 ± 1.1*^,†^	−3.1 ± 0.6*^,†,‡^

NLX, naloxone hydrochloride (1.5 mg/kg, IV). MAP, mean arterial blood pressure. D-CYSee, D-cysteine ethyl ester (250 μmol/kg, IV). D-CYSea, D-cysteine ethyl amide (100 μmol/kg, IV). The dose of D-cysteine was 250 μmol/kg, IV. The data are shown as mean ± SEM. There were 9 rats in each group. *p < 0.05, significant response from Pre. ^†^ *p* < 0.05, D-CYSea or D-CYSee versus vehicle. ^‡^ *p* < 0.05, D-CYSea versus D-CYSee.

**TABLE 2:** Body temperatures (^o^C) at key points of each of the study protocols.

Co-injection 1–5 Study	Body temperature (^o^C) and arithmetic changes in temperature			
	Vehicle	D-cysteine	D-CYSee	D-CYSea
Pre	37.5 ± 0.06	37.5 ± 0.07	37.5 ± 0.06	37.5 ± 0.06
Post-inj 5	38.1 ± 0.07	38.2 ± 0.11	37.5 ± 0.08	37.6 ± 0.08
Post-NLX	37.3 ± 0.11	37.3 ± 0.17	37.3 ± 0.08	37.5 ± 0.12
Post-inj 5 vs. Pre	+0.56 ± 0.07*	+0.70 ± 0.06*	+0.04 ± 0.05^†^	+0.12 ± 0.06^†^
Post-NLX vs. Post-inj 5	−0.76 ± 0.09*	−0.89 ± 0.10*	−0.17 ± 0.06*^,†^	−0.14 ± 0.6^†^
Co-injection 1–10 Study	Vehicle	D-cysteine	D-CYSee	D-CYSea
Pre	37.6 ± 0.08	37.5 ± 0.06	37.4 ± 0.07	37.5 ± 0.07
Post-inj 10	38.3 ± 0.08	38.3 ± 0.07	37.5 ± 0.11	37.5 ± 0.04
Post-NLX	36.6 ± 0.19	36.6 ± 0.21	37.4 ± 0.11	37.4 ± 0.10
Post-inj 10 vs. Pre	+0.72 ± 0.08*	+0.80 ± 0.10*	+0.12 ± 0.06^†^	+0.02 ± 0.07^†^
Post-NLX vs. Post-inj 10	−1.66 ± 0.17*	−1.77 ± 0.17*	−0.10 ± 0.9^†^	−0.08 ± 0.08^†^
Co-injection 6–10 Study	Vehicle	D-cysteine	D-CYSee	D-CYSea
Pre	37.5 ± 0.07	37.5 ± 0.09	37.5 ± 0.08	37.5 ± 0.08
Post-inj 10	38.4 ± 0.13	38.4 ± 0.13	37.7 ± 0.10	37.7 ± 0.07
Post-NLX	36.6 ± 0.2	36.5 ± 0.13	37.2 ± 0.11	37.5 ± 0.09
Post-inj 10 vs. Pre	+0.92 ± 0.09*	+0.89 ± 0.11*	+0.22 ± 0.04*^,†^	+0.17 ± 0.07*^,†^
Post-NLX vs. Post-inj 10	−1.84 ± 0.11*	−1.94 ± 0.14*	−0.48 ± 0.06*^,†^	−0.18 ± 0.5*^,†,‡^

NLX, naloxone hydrochloride (1.5 mg/kg, IV). MAP, mean arterial blood pressure. D-CYSee, D-cysteine ethyl ester (250 μmol/kg, IV). D-CYSea, D-cysteine ethyl amide (100 μmol/kg, IV). The dose of D-cysteine was 250 μmol/kg, IV. The data are shown as mean ± SEM. There were 9 rats in each group. *p < 0.05, significant response from Pre. ^†^ *p* < 0.05, D-CYSea, or D-CYSee, versus vehicle. ^‡^ *p* < 0.05, D-CYSea versus D-CYSee.

MAP, heart rate, and Heart Rate/MAP values before and after injection of NLX, and arithmetic changes in these parameters, for co-injections 1–5, co-injections 1–10, and co-injections 6–10 studies are presented in Table 3. There were no between-group differences in resting parameters prior to the administration of NLX (the injections of fentanyl elicited transient decreases in MAP and heart rate that had fully resolved by the time the pre-NLX values were recorded). The NLX-precipitated increases in MAP and heart rate in rats that received 10 co-injections of fentanyl + vehicle were substantially greater than in rats given 5 co-injections of fentanyl + vehicle. The NLX-precipitated increases in MAP and heart rate were smaller in rats that received fentanyl + D-CYSee and fentanyl + D-CYSea in co-injections 1–5, co-injections 1–10, and co-injections 6–10 studies. Arithmetic changes in Heart Rate/ MAP values (see column Delta) were enhanced in rats that received co-injections 1–10 and co-injections 6–10 of fentanyl + vehicle. These ratios were markedly diminished in rats that received co-injections of fentanyl + D-CYSee or fentanyl + D-CYSea for all three studies.

**TABLE 3:** Cardiorespiratory responses elicited by the injection of naloxone HCl.

Study	MAP (mmHg)			Heart rate (beats/min)			Heart rate/MAP (bpm/mmHg)		
Co-inj 1–5	Pre	Post-NLX	Delta	Pre	Post-NLX	Delta	Pre	Post-NLX	Delta
Vehicle	113 ± 2	129 ± 2	+16.3 ± 1.7*	355 ± 5	410 ± 6	+54.1 ± 4.0*	3.16 ± 0.07	3.18 ± 0.07	3.64 ± 0.52*
D-cysteine	114 ± 1	136 ± 3	+21.3 ± 2.2*	355 ± 5	423 ± 7	+68.0 ± 9.1*	3.10 ± 0.06	3.12 ± 0.06	3.40 ± 0.51*
D-CYSee	115 ± 1	121 ± 2	+5.1 ± 0.7*^,†^	358 ± 8	365 ± 9	+6.9 ± 1.6*^,†^	3.10 ± 0.07	3.03 ± 0.07	1.35 ± 0.22*^,†^
D-CYSea	115 ± 2	120 ± 2	+4.6 ± 0.7*^,†^	357 ± 5	365 ± 5	+7.9 ± 1.8*^,†^	3.12 ± 0.05	3.05 ± 0.06	1.98 ± 0.41*^,†^
Co-inj 1–10	Pre	Post-NLX	Delta	Pre	Post-NLX	Delta	Pre	Post-NLX	Delta
Vehicle	114 ± 2	144 ± 4	+30.3 ± 3.3*	357 ± 6	478 ± 12	+121.2 ± 10.3*	3.14 ± 0.06	3.33 ± 0.07	4.32 ± 0.52*
D-cysteine	114 ± 2	151 ± 4	+36.4 ± 3.9*	356 ± 7	512 ± 9	+155.6 ± 12.6*	3.12 ± 0.06	3.41 ± 0.08	4.61 ± 0.50*
D-CYSee	112 ± 2	121 ± 2	+8.4 ± 1.4*^,†^	358 ± 6	366 ± 6	+8.7 ± 2.3*^,†^	3.19 ± 0.06	3.04 ± 0.06	1.12 ± 0.18*^,†^
D-CYSea	114 ± 1	118 ± 2	+3.6 ± 0.5*^,†^	357 ± 4	362 ± 4	+4.9 ± 0.5*^,†^	3.13 ± 0.05	3.08 ± 0.05	1.56 ± 0.22*^,†^
Co-inj 6–10	Pre	Post-NLX	Delta	Pre	Post-NLX	Delta	Pre	Post-NLX	Delta
Vehicle	115 ± 1	150 ± 5	+34.9 ± 4.1*	358 ± 5	496 ± 15	+138.7 ± 14.4*	3.11 ± 0.04	3.31 ± 0.06	4.25 ± 0.40*
D-cysteine	116 ± 1	153 ± 5	+37.1 ± 4.3*	354 ± 5	508 ± 13	+154.3 ± 15.7*	3.06 ± 0.05	3.32 ± 0.06	4.19 ± 0.24*
D-CYSee	112 ± 1	123 ± 2	+10.8 ± 1.3*^,†^	358 ± 5	386 ± 7	+28.1 ± 6.3*^,†^	3.20 ± 0.06	3.15 ± 0.06	2.51 ± 0.36*^,†^
D-CYSea	114 ± 1	118 ± 2	+3.4 ± 0.5*^,†,‡^	360 ± 5	368 ± 6	+7.8 ± 1.7*^,†,‡^	3.16 ± 0.08	3.13 ± 0.08	2.24 ± 0.38*^,†^

MAP, mean arterial blood pressure. bpm, beats per minute. NLX, naloxone hydrochloride (1.5 mg/kg, IV). D-CYSee, D-cysteine ethyl ester (250 μmol/kg, IV). D-CYSea, D-cysteine ethyl amide (100 μmol/kg, IV). The dose of D-cysteine was 250 μmol/kg, IV. The data are shown as mean ± SEM. There were 9 rats in each group. *p < 0.05, significant response from Pre. ^†^ *p* < 0.05, D-CYSea or D-CYSee versus vehicle. ^‡^ *p* < 0.05, D-CYSea versus D-CYSee.

## Discussion

We show here that the injection of NLX elicited pronounced withdrawal syndrome consisting of behavioral and cardiorespiratory responses, and falls in body temperature and body weight, in male rats that received twice-daily co-injections of fentanyl (125 μg/kg, IV) + vehicle. These withdrawal phenomena seen in this study are strongly suggestive of the rats having become physically-dependent on fentanyl, and are consistent with reports on the patterns of NLX-precipitated withdrawal signs seen in a variety of fentanyl-administration protocols (Bartoletti et al., 1987; Adams and Wooten, 1994; De Kock and Meert, 1995; Thornton and Smith, 1997; Fendt and Mucha, 2001; Lohmann and Smith, 2001; Chen and Pan, 2006; Bruijnzeel et al., 2007; Liu et al., 2008; Mitzelfelt et al., 2011; Mitzelfelt et al., 2014; Gyawali et al., 2020; Uddin et al., 2021), and in other protocols used to induce physical dependence to opioids (Laska et al., 1976; Laska et al., 1977; Hutchinson et al., 2007; Lopez-Gimenez and Milligan, 2010; Morgan and Christie, 2011; Nielsen and Kreek, 2012). Moreover, the lack of behavioral responses elicited by the injection of NLX in rats that received co-injections of vehicle + vehicle, vehicle + D-CYSee or vehicle + D-CYSea suggests that the behavioral phenomena that occurred upon the injection of NLX in fentanyl-injected rats were indeed withdrawal phenomena (due to the development of physical dependence to the opioid), rather than other potential issues (e.g., multiple injection protocols) that would lead NLX to cause behavioral reactions. The NLX-induced hypertension and tachycardia are consistent with previous studies showing that NLX-precipitated withdrawal is associated with hypertension and tachycardia due to activation of the sympathetic nervous system in humans (Purssell et al., 1995; Walsh et al., 2003; Levin et al., 2019; Balshaw et al., 2021; Baldo, 2022; Isoardi et al., 2022; Lee et al., 2022) and experimental animals (Buccafusco, 1983; Buccafusco, 1990; Buccafusco et al., 1984; Marshall and Buccafusco, 1985; Dixon and Chang, 1988; Chang and Dixon WR, 1990; Delle et al., 1990; Baraban et al., 1993). Our observation that the NLX produced a marked increase in apneas (>1.5 s between breaths) is also in agreement with results from opioid withdrawal paradigms in humans (Schwarzer et al., 2015; Zamani et al., 2020; Wilson et al., 2023) and rats (Delle et al., 1990; Baldo, 2022).

The first new conclusion of this study was that co-injections of D-CYSee and D-CYSea reduced the development of physical dependence to fentanyl. This conclusion was based on the findings that the withdrawal phenomena elicited by NLX (e.g., behavioral changes, elevations in MAP and heart rate, and falls in body weight and temperature) were substantially less than in rats that were co-injected with fentanyl + vehicle. The finding that D-cysteine was ineffective suggests that the cell-penetrability of D-CYSee and D-CYSea is an important factor in their efficacy. The enhanced potency of D-CYSea over D-CYSee may result from greater resistance to plasma carboxylesterases that convert thiol esters, such as D-CYSee, to parent thiols (Butterworth et al., 1993; Nishida et al., 1996). As such more D-CYSea than D-CYSee may enter brain neurons involved in acquisition of physical dependence and addiction (Laschka et al., 1976a; Laschka et al., 1976b; Laschka et al., 1977; Koob, 1987; Saiepour et al., 2001; Glass, 2010; Gardner, 2011). However, it should be noted that although a lower dose of D-CYSea (100 μmol/kg, IV) was more effective than a higher dose of D-CYSee (250 μmol/kg, IV) in preventing the development of dependence (markedly reduced NLX-precipitated withdrawal phenomena), we observed that D-CYSea markedly diminished NLX-precipitated sneezing, whereas D-CYSee did not. It is well-known that sneezing is an important phenomenon in opioid withdrawal syndromes in humans (Ostrea et al., 1975; Specker et al., 1998; Gaalema et al., 2012; Lofwall et al., 2013) and animals (Hendrie, 1985; Liu et al., 2007). One possibility therefore is that D-CYSea interacts with signaling pathways driving sneezing (Batsel and Lines, 1975; Undem et al., 2000; Li et al., 2021; Ramirez et al., 2022), whereas D-CYSee does not. The activity of D-CYSea raises the possibility that this, and other ethyl amides, such as the antioxidant N-acetyl-L-cysteine (L-NAC) ethyl amide (Bahat-Stroomza et al., 2005; Grinberg et al., 2005; Ates et al., 2008; Annia, 2011; Sunitha et al., 2013), may show efficacy in human trials.

Currently, we have no direct evidence as to the cellular mechanisms by which D-CYSee and D-CYSea blunt the development of physical dependence to fentanyl. Previous research suggests that the mechanisms may involve 1) their reducing potential (e.g., reduction of Fe^3+^ to Fe^2+^, free L-cystine to L-cysteine), and reduction of protein bound L-cystine to L-cysteine in plasma membrane ion-channels, including K^+^-, Ca^2+^- and non-selective cation channels (Baronas et al., 2017; Ward, 2017; Weise-Cross et al., 2019), and major ligand-gated ion channel receptors, such as N-methyl-D-aspartate (NMDA) glutamatergic receptors (Sucher and Lipton, 1991) and γ-aminobutyric acid (GABA) receptors (Calvo et al., 2016), 2) their redox regulation of an array of functional proteins upon entering cells (Bogeski et al., 2011; Bogeski and Niemeyer, 2014; O-Uchi et al., 2014; Gamper and Ooi, 2015; Gao et al., 2017; García et al., 2018), 3) conversion of D-CYSee and D-CYSea to D-cysteine which then enters into enzymatic processes generating H_2_S sequentially by D-aminoacid oxidase and 3-mercaptopyruvate sulfur-transferase (Kimura, 2014; 2017; Bełtowski, 2019) in cells, such as the carotid bodies (Prabhakar, 2012), and 4) conversion to S-nitroso-D-CYSee and S-nitroso-D-CYSea, which exert effects similar to endogenous S-nitrosothiols, such as S-nitroso-L-cysteine (Myers et al., 1990; Bates et al., 1991; Seckler et al., 2017; 2020), which controls various intracellular processes (Lipton et al., 1993; Foster et al., 2009; Seth and Stamler, 2011; Stomberski et al., 2019; Gaston et al., 2020), including those that control cardiorespiratory functions (Davisson et al., 1996; 1997; Ohta et al., 1997; Lipton et al., 2001; Gaston et al., 2006; Lewis et al., 2006), and those diminishing opioid-induced respiratory depression (OIRD) (Getsy et al., 2022d; 2022e). These actions of D-CYSee and D-CYSea may affect the cell-signaling processes thought to be important in acquisition of physical dependence to opioids, including those involving NMDA receptors (Buccafusco et al., 1995; Herman et al., 1995; Rasmussen, 1995; Noda and Nabeshima, 2004; Glass, 2011; Fluyau et al., 2020), muscarinic receptors (Marshall and Buccafusco, 1985; Holland et al., 1993), voltage-gated Ca^2+^-channels (Tokuyama et al., 1995; Dogrul et al., 2002; Esmaeili-Mahani et al., 2008; Alboghobeish et al., 2019), oxidative stress (Mori et al., 2007; Abdel-Zaher et al., 2013; Mansouri et al., 2020; Ward et al., 2020; Houshmand et al., 2021), and the nitric oxide-initiated cGMP-mediated signaling cascades (Adams et al., 1993; Cappendijk et al., 1993 Majeed et al., 1994; Leza et al., 1995; 1996; London et al., 1995; Vaupel et al., 1995a; Vaupel et al., 1995b; Dambisya and Lee, 1996; Bhatt and Kumar, 2015; Tsakova et al., 2015; Sackner et al., 2019; Gledhill and Babey, 2021). The redox effects of D-CYSee and D-CYSea are also likely important since a primary intracellular redox regulator, α-lipoic acid, diminishes the development of morphine dependence in mice and the NLX-induced biochemical alterations in morphine-dependent mice. Moreover, the actions of α-lipoic acid were enhanced by concurrent treatment with N-acetyl-L-cysteine (Abdel-Zaher et al., 2013).

The second new conclusion from this study was that co-injections of D-CYSee and D-CYSea beginning with the 6th and continuing to the 10th injection of fentanyl, reverse established physical dependence to the opioid (again on the basis of diminished NLX-precipitated responses). The NLX-precipitated behaviors (except for sneezes), increases in MAP, heart rate, and the numbers of apneas, and falls in body temperature and body weight, were fewer or smaller in magnitude in rats that received the co-injections of D-CYSee. Notably, the NLX-precipitated withdrawal phenomena, including sneezes, were markedly attenuated in rats that received co-injections of D-CYSea. Again, the mechanisms by which D-CYSee and D-CYSea reverse acquired physical dependence to fentanyl are unknown, however, some of the mechanisms discussed above, including their antioxidant properties, may be involved. Although none of the following have proven to be tenable therapeutics, the drugs and agents that have some ability to reverse established physical dependence include, the antioxidants, quercetin and melatonin; allosteric modulators of AMPA receptors; the dopamine D2 receptor antagonist, haloperidol; adrenomedullin receptor antagonists; the β_2_-AR antagonist, butoxamine; histamine receptor agonists; a 5-hydroxytryptamine-reuptake inhibitor, fluoxetine; inhibitors of Ca^2+^/calmodulin-dependent protein kinase II; and the nitric oxide synthase inhibitor, L-N^G^-nitro-arginine methyl ester. The clear lack of rationale for the use of these drugs and agents speaks to our minimal understanding about mechanisms underlying physical dependence to opioids. Our findings that D-CYSee and D-CYSea reverse established physical dependence to fentanyl, and our finding that D-CYSee prevents the development of fentanyl-induced conditioned place preference (addictive potential) in male and female rats (Knauss et al., 2023), certainly supports the concept that alterations in thiol chemistry within cells may be an important common feature of fentanyl-induced addiction and physical dependence (Trivedi et al., 2014). The ability of D-CYSee and D-CYSea to reverse established physical dependence to fentanyl has potential clinical relevance given that these, and other bioactive L,D-thiol esters/amides (Gaston et al., 2021; Jenkins et al., 2021; Getsy et al., 2022a; Getsy et al., 2022b; Getsy et al., 2022c; Getsy et al., 2022d; Lewis et al., 2022) and related compounds, such as L-NAC (Getsy et al., 2022f), Tempol (Baby et al., 2021a, Baby et al., 2021b), and S-nitrosothiols (Getsy et al., 2022d; Getsy et al., 2022e), have been shown to be able to reverse acquired physical dependence to fentanyl and other opioids in humans. In particular, if D-CYSee and D-CYSea, for example, can block opioid-induced dopamine surges in brain structures (e.g., medial prefrontal cortex, ventral tegmentum and nucleus accumbens) in which the rewarding euphoria-producing dopamine surge happens for drugs of abuse/addiction (Laschka et al., 1976a; Laschka et al., 1976b; Laschka et al., 1977; Koob, 1987; Saiepour et al., 2001; Glass, 2010; Gardner, 2011), then they could be useful in treatment of opioid use disorder (OUD) as stand-alone therapies. Moreover, maternal opioid use is a fast-growing public health issue, and babies born to mothers dependent on opioids often display severe withdrawal symptoms that require hospitalization (Kelly et al., 2020; Centers for Disease Control and Prevention, 2023). Current treatment strategies of this neonatal opioid withdrawal syndrome (NOWS) are inadequate, and the infants develop numerous behavioral, and cognitive social problems as they grow older (Winklbaur et al., 2008; Jones et al., 2010; Reddy et al., 2017). As such, novel therapies and better understanding of the mechanisms by which drugs and agents benefit the immediate and long-term consequences of NOWS are desperately needed. Recent compelling findings with L-NAC and L-NAC methyl ester suggest that they may be of therapeutic benefit in preventing the development of NOWS.

### Limitations

While this study demonstrates the efficacies of D-CYSee and D-CYSea in male rats, it is imperative to determine whether these compounds prevent and reverse physical dependence to fentanyl in female rats. It is well known that there are numerous sex-dependent differences in opioid receptor signaling (Bryant et al., 2006; Hosseini et al., 2011), and that opioids exert qualitatively/quantitatively different pharmacological responses (e.g., cardiorespiratory and antinociceptive) in females compared to males (Dahan et al., 1998; Sarton et al., 1998; Bodnar and Kest, 2010). Moreover, there are marked sex-dependent differences in expression of opioid-induced hyperalgesia, tolerance and withdrawal (Bodnar and Kest, 2010), and several sex differences in the expression of OUDs (Knauss et al., 2023) and treatment strategies for these disorders (Huhn et al., 2019; Davis et al., 2021; Knouse and Briand, 2021). Additionally, we must perform studies with lower doses of L-CYSee and D-CYSea in both and male rats to better understand their efficacy and potential side-effect profiles. For example, we have yet to establish whether co-injections of D-CYSee and D-CYSea alters fentanyl-induced changes in antinociception status (e.g., analgesia and, hyperalgesia), although we have found that injections of D- or L-cysteine (m) ethyl esters (Getsy et al., 2022a; Getsy et al., 2022b; Getsy et al., 2022c; Getsy et al., 2022d; Lewis et al., 2022), D-cysteine di (m) ethyl ester (Gaston et al., 2021), L-glutathione ethyl ester (Jenkins et al., 2021), and L-NAC (Getsy et al., 2022f) do not impair the analgesia induced by opioids despite preventing/reversing the OIRD. Our lack of knowledge as to cellular/molecular mechanisms of action of D-CYSee and D-CYSea hinders our understanding of how they can exert their effects. Our efforts to date are focused on possible binding of D-CYSee and D-CYSea, and the parent thiol, D-cysteine, to L,D-cysteine binding protein myristoylated alanine-rich C-kinase substrate (Semenza et al., 2021). Additionally, we are focused on establishing whether disruption of opioid receptor-β-arrestin cell signaling events mediating the development of physical dependence, while sparing the G-protein-mediated analgesic effects of opioids (Schmid et al., 2017; Grim et al., 2020), may involve the conversion of D-CYSee and D-CYSea to their S-nitrosothiol forms which then drives their activity. This theory is based on 1) evidence that the S-nitrosothiol, S-nitroso-L-cysteine, overcomes fentanyl- and morphine-induced OIRD (Getsy et al., 2022d; Getsy et al., 2022e), 2) the bioactivity of S-nitroso-L-cysteine ethyl ester (Clancy et al., 2001), and 3) evidence that oral S-nitroso-L-NAC methyl ester has unique pharmacological properties associated with the generation of intracellular glutathione, H_2_S, and nitrosyl entities (Tsikas et al., 2018). With respect to understanding whether D-CYSee and D-CYSea penetrate into brain structures involved in acquisition of opioid dependence, we are currently determining the plasma and brain distribution of these compounds upon systemic injection with and without co-injections of fentanyl via LC-MS (Altawallbeh et al., 2019). With the rapidly expanding role of synthetic opioids in the major OUD crisis (Arendt, 2021; Deo et al., 2021), our studies must ultimately determine whether D-CYSee and/or D-CYSea overcome physical dependence to fentanyl in humans. One final consideration in addition to testing if the effects of D-CYSee and D-CYSea generalize across sexes, is to determine if the results translate within species. Decades of work in mice and rats have shown that opioid withdrawal phenotypes are genetically heritable traits subject to genetic variation (Kest et al., 2004; Philip et al., 2010). The lack of genetic variation in preclinical models is one explanation for the inability of many findings to translate across species (Garner, 2014; Zuberi et al., 2016). Additionally, pre-clinical screening of therapeutics has largely ignored multigenic effects by testing inbred rodent strains—devoid of genetic variation—and manipulating individual gene mutations that do not accurately recapitulate human disease pathophysiology (Mosedale, 2018). Testing the efficacy of drugs using a population of outbred mice containing approximately 45 million segregating single nucleotide polymorphisms (SNPs), such as the Diversity Outbred (Saul et al., 2019; Li and Auwerx, 2020), that has diversity similar to what is found in the human population, will increase the likelihood that the drug translates within species before traversing across species.

## Conclusion

Using the degree of NLX-induced withdrawal phenomena as a measure, we show here that D-CYSee and D-CYSea prevent the development of physical dependence to fentanyl, and reverse acquired dependence to this synthetic opioid in adult male Sprague Dawley rats. The enhanced efficacy of D-CYSea is potentially due to its greater resistance to carboxylesterases that may convert D-CYSee to D-cysteine. Our study with D-CYSee and D-CYSea was in large part due to the pioneering work of Trivedi and Deth (2015) and Trivedi et al. (2014) which greatly contributed to our knowledge about the cell processes by which opioids cause addiction and dependence (Getsy et al., 2022a). In particular, the possibility that opioids may cause psychological addiction and physical dependence by opioid-receptor-mediated blockade of EAAT3/EAAC1 transporter-mediated entry of L-cysteine into neurons (Trivedi et al., 2014), prompted our pharmacological studies with the membrane-permeable, L-cysteine ethyl ester, as well as D-CYSee and D-CYSea, and other D,L-thiol esters. The findings that D-CYSee and D-CYSea both markedly reduced the larger majority of NLX-precipitated withdrawal phenomena, suggests that the loss of L-cysteine entry into cells plays a key role in establishing physical dependence to fentanyl. The enhanced potency of D-CYSea compared to D-CYSee points to an important strategy in the development of therapeutically effective thiol drugs that not only brings enhanced cell-penetrability, but also potential protection from plasma carboxylesterases. Determining the thiol-dependent signaling pathways by which D-CYSee and D-CYSea exert their therapeutic actions will add to our understanding how these two classes of thiol analogues exert their effects.

## Data Availability

The raw data supporting the conclusion of this article will be made available by the authors, without undue reservation.
