# Supplementary material for: Lipophilic analogues of D-cysteine prevent and reverse physical dependence to fentanyl in male rats
Source: Front Pharmacol. 2024 Apr 5;14:1336440. doi: 10.3389/fphar.2023.1336440 (PMC11026688; doi:10.3389/fphar.2023.1336440)
Supplement: Supplementary file 1 [file Table1.docx]

**Supplementary File**

**Lipophilic analogues of D-cysteine prevent and reverse**

**physical dependence to fentanyl in male rats**

James N. Bates,^1,†^ Paulina M. Getsy,^2^ Gregory A. Coffee,^2^

Santhosh M. Baby,^3,‡^ Peter M. MacFarlane,^2^ Yee-Hsee Hsieh,^4^ Zackery T. Knauss,^5^

Jason A. Bubier,^6^ Devin Mueller^5^ and Stephen J. Lewis^2,7,8,^*

*^1^Department of Anesthesiology, University of Iowa Hospitals and Clinics, Iowa City, Iowa, USA*

*^2^Department of Pediatrics, Case Western Reserve University, Cleveland, Ohio, USA*

*^3^Section of Biology, Galleon Pharmaceuticals, Inc., Horsham, Pennsylvania, USA*

*^4^Division of Pulmonary, Critical Care and Sleep Medicine, Case Western Reserve University,*

*Cleveland, Ohio, USA*

*^5^Department of Biological Sciences, Kent State University, Kent, Ohio, USA*

*^6^The Jackson Laboratory, Bar Harbor, Maine, USA*

*^7^Department of Pharmacology,* *Case Western Reserve University, Cleveland, Ohio, USA*

*^8^Functional Electrical Stimulation Center, Case Western Reserve University, Cleveland, Ohio, USA*

**^†^Present Address:** James N. Bates, Chief Medical Officer, *Atelerix Life Sciences Inc*. Address: 300 East Main Street, Suite 202 Charlottesville, Virginia 22902. Email: jbates@atelerixlifesciences.com. https://atelerixlifesciences.com

**^‡^Present address:** Translational Sciences Treatment Discovery, Galvani Bioelectronics, Inc, 1250 S Collegeville Rd, Collegeville, PA 1r9426, USA. Email: babysanthosh@gmail.com

***Corresponding Author:** Stephen J. Lewis, PhD. Department of Pediatrics, Division of Pulmonology, Allergy and Immunology, School of Medicine, Case Western Reserve University, 10900 Euclid Avenue, Cleveland, OH 44106-4984. Email: sjl78@case.edu

**Supplementary Table S1**

Body weights for each group of rats

|  |  |  |  |  |  | **Treatment Groups** | | | | | | |
| --- | --- | --- | --- | --- | --- | --- | --- | --- | --- | --- | --- | --- |
| **Study** |  | **Test** |  | **Protocol** |  | **Vehicle** |  | **D-CYSee** |  | **D-CYSea** |  | **D-Cysteine** |
| **A. Fentanyl groups** |  | Behaviors |  | Inj1-5 |  | 337 ± 1.7 |  | 337 ± 1.8 |  | 337 ± 1.4 |  | 337 ± 1.3 |
| 48 groups, 9 rats per group |  |  |  | Inj1-10 |  | 337 ± 1.0 |  | 337 ± 1.6 |  | 338 ± 2.1 |  | 340 ± 2.1 |
| 416 rats in total |  |  |  | Inj6-10 |  | 333 ± 1.0 |  | 335 ± 1.5 |  | 334 ± 1.1 |  | 335 ± 0.9 |
|  |  | MAP, HR |  | Inj1-5 |  | 338 ± 1.6 |  | 337 ± 1.6 |  | 337 ± 1.8 |  | 339 ± 2.3 |
|  |  |  |  | Inj1-10 |  | 337 ± 2.0 |  | 337 ± 2.3 |  | 338 ± 1.6 |  | 337 ± 1.7 |
|  |  |  |  | Inj6-10 |  | 335 ± 1.3 |  | 334 ± 1.8 |  | 334 ± 1.0 |  | 335 ± 1.5 |
|  |  | Apneas |  | Inj1-5 |  | 337 ± 2.4 |  | 337 ± 1.3 |  | 336 ± 1.6 |  | 339 ± 2.3 |
|  |  |  |  | Inj1-10 |  | 334 ± 1.8 |  | 336 ± 1.7 |  | 336 ± 1.8 |  | 336 ± 1.9 |
|  |  |  |  | Inj6-10 |  | 339 ± 2.4 |  | 338 ± 1.7 |  | 336 ± 1.5 |  | 337 ± 1.5 |
|  |  | BW, BT |  | Inj1-5 |  | 338 ± 1.5 |  | 337 ± 1.5 |  | 338 ± 1.4 |  | 338 ± 1.7 |
|  |  |  |  | Inj1-10 |  | 339 ± 1.4 |  | 339 ± 2.0 |  | 341 ± 1.3 |  | 337 ± 1.6 |
|  |  |  |  | Inj6-10 |  | 338 ± 1.6 |  | 340 ± 2.5 |  | 336 ± 1.6 |  | 337 ± 2.0 |
| **B. Vehicle groups** |  | Behaviors |  | Inj1-5 |  | 335 ± 2.0 |  | 334 ± 1.7 |  | 336 ± 1.6 |  | ND |
| 18 groups |  |  |  | Inj1-10 |  | 334 ± 1.4 |  | 335 ± 1.6 |  | 335 ± 1.7 |  | ND |
| 9 rats per group |  |  |  | Inj6-10 |  | 335 ± 1.7 |  | 335 ± 1.7 |  | 336 ± 1.7 |  | ND |
| 162 rats in total |  | BW, BT |  | Inj1-5 |  | 334 ± 1.4 |  | 335 ± 1.1 |  | 337 ± 1.9 |  | ND |
|  |  |  |  | Inj1-10 |  | 337 ± 1.0 |  | 336 ± 1.8 |  | 336 ± 2.0 |  | ND |
|  |  |  |  | Inj6-10 |  | 336 ± 1.7 |  | 335 ± 1.2 |  | 335 ± 1.9 |  | ND |

ND, not determined. D-CYSee, D-cysteine ethyl ester. D-CYSea, D-cysteine ethyl amide. MAP, mean arterial blood pressure. HR, heart rate. BW, body weight. BT, body temperature. The data are shown as mean ± SEM. There were 9 rats in each group. There were no between group differences for any body weight value (*p* > 0.05, for all comparisons).

**Supplementary Table S2**

Behavioral responses elicited by NLX in rats that received multiple co-injections of vehicle + vehicle, vehicle + D-CYSee or vehicle + D-CYSea

|  |  |  |  | **NLX-precipitated withdrawal behaviors** | | | | | | | | | | | | |
| --- | --- | --- | --- | --- | --- | --- | --- | --- | --- | --- | --- | --- | --- | --- | --- | --- |
| **Injections** |  | **Agent** |  | **Jumps** |  | **WDS** |  | **Rears** |  | **FPL** |  | **Circles** |  | **Sneezes** |  | **Writhes** |
| **Inj1-5** |  | Vehicle |  | 0.6 ± 0.3 |  | 0.6 ± 0.3 |  | 0.8 ± 0.4 |  | 0.6 ± 0.3 |  | 0.0 ± 0.0 |  | 0.7 ± 0.4 |  | 0.2 ± 0.1 |
|  |  | D-CYSee |  | 0.2 ± 0.1 |  | 0.1 ± 0.1 |  | 0.7 ± 0.3 |  | 0.9 ± 0.4 |  | 0.1 ± 0.1 |  | 0.4 ± 0.3 |  | 0.0 ± 0.0 |
|  |  | D-CYSea |  | 0.0 ± 0.0 |  | 0.1 ± 0.1 |  | 0.9 ± 0.8 |  | 0.7 ± 0.4 |  | 0.0 ± 0.0 |  | 0.2 ± 0.1 |  | 0.2 ± 0.1 |
| **Inj1-10** |  | Vehicle |  | 0.0 ± 0.0 |  | 0.3 ± 0.2 |  | 0.4 ± 0.2 |  | 0.9 ± 0.4 |  | 0.2 ± 0.2 |  | 0.7 ± 0.4 |  | 0.1 ± 0.1 |
|  |  | D-CYSee |  | 0.2 ± 0.1 |  | 0.0 ± 0.0 |  | 0.4 ± 0.3 |  | 0.9 ± 0.5 |  | 0.0 ± 0.0 |  | 0.9 ± 0.6 |  | 0.0 ± 0.0 |
|  |  | D-CYSea |  | 0.0 ± 0.0 |  | 0.2 ± 0.2 |  | 0.8 ± 0.4 |  | 1.2 ± 0.6 |  | 0.0 ± 0.0 |  | 0.8 ± 0.5 |  | 0.0 ± 0.0 |
| **Inj6-10** |  | Vehicle |  | 0.7 ± 0.4 |  | 0.3 ± 0.2 |  | 0.8 ± 0.6 |  | 1.3 ± 0.6 |  | 0.4 ± 0.3 |  | 1.2 ± 0.7 |  | 0.0 ± 0.0 |
|  |  | D-CYSee |  | 0.0 ± 0.0 |  | 0.4 ± 0.2 |  | 0.7 ± 0.5 |  | 1.3 ± 0.6 |  | 0.6 ± 0.2 |  | 0.6 ± 0.4 |  | 0.0 ± 0.0 |
|  |  | D-CYSea |  | 0.1 ± 0.1 |  | 0.2 ± 0.2 |  | 0.3 ± 0.2 |  | 1.0 ± 0.5 |  | 0.1 ± 0.1 |  | 0.7 ± 0.4 |  | 0.1 ± 0.1 |

D-CYSee, D-cysteine ethyl ester. D-CYSea, D-cysteine ethyl amide. WDA, wet-dog shaking. FPL, fore-paw licking. The data are shown as mean ± SEM. There were 9 rats in each group. There were no between group differences for any body weight value (*p* > 0.05, for all comparisons).

**Supplementary Table S3**

Body temperatures and body weights at key points of control (i.e., vehicle, no fentanyl) study

| **Parameter** |  | **Injections** |  | **Agent** |  | **Pre-Inj1** |  | **Pre-NLX** |  | **Post-NLX** |  | **Pre-NLX versus**  **Pre-Inj1** |  | **Post-NLX versus Pre-NLX** |
| --- | --- | --- | --- | --- | --- | --- | --- | --- | --- | --- | --- | --- | --- | --- |
| **BW, gram** |  | Inj1-5 |  | Vehicle |  | 334 ± 1.4 |  | 337 ± 1.4 |  | 336 ± 1.2 |  | +2.1 ± 0.5* |  | -1.0 ± 0.5 |
|  |  |  |  | D-CYSee |  | 335 ± 1.1 |  | 338 ± 0.9 |  | 338 ± 1.2 |  | +3.6 ± 0.4* |  | -0.3 ± 0.6 |
|  |  |  |  | D-CYSea |  | 337 ± 1.9 |  | 340 ± 1.8 |  | 340 ± 1.4 |  | +3.2 ± 0.5* |  | -0.3 ± 0.5 |
|  |  | Inj1-10 |  | Vehicle |  | 337 ± 2.0 |  | 343 ± 1.7 |  | 343 ± 2.1 |  | +6.1 ± 0.7* |  | -0.7 ± 0.7 |
|  |  |  |  | D-CYSee |  | 336 ± 1.8 |  | 342 ± 1.6 |  | 342 ± 1.3 |  | +6.6 ± 0.6* |  | -0.8 ± 0.7 |
|  |  |  |  | D-CYSea |  | 336 ± 2.0 |  | 343 ± 2.3 |  | 342 ± 1.9 |  | +6.9 ± 0.8* |  | -0.9 ± 0.8 |
| **BT, ^o^C** |  | Inj1-5 |  | Vehicle |  | 37.5 ± 0.06 |  | 37.5 ± 0.05 |  | 37.6 ± 0.08 |  | +0.04 ± 0.08 |  | +0.11 ± 0.06 |
|  |  |  |  | D-CYSee |  | 37.5 ± 0.06 |  | 37.5 ± 0.07 |  | 37.5 ± 0.08 |  | -0.04 ± 0.06 |  | +0.02 ± 0.08 |
|  |  |  |  | D-CYSea |  | 37.4 ± 0.05 |  | 37.4 ± 0.06 |  | 37.5 ± 0.08 |  | 0.00 ± 0.06 |  | +0.06 ± 0.06 |
|  |  | Inj1-10 |  | Vehicle |  | 37.5 ± 0.06 |  | 37.5 ± 0.04 |  | 36.6 ± 0.05 |  | +0.04 ± 0.06 |  | +0.08 ± 0.07 |
|  |  |  |  | D-CYSee |  | 37.6 ± 0.06 |  | 37.6 ± 0.05 |  | 37.7 ± 0.06 |  | +0.02 ± 0.07 |  | +0.02 ± 0.06 |
|  |  |  |  | D-CYSea |  | 37.6 ± 0.06 |  | 37.5 ± 0.06 |  | 37.6 ± 0.09 |  | -0.01 ± 0.06 |  | +0.07 ± 0.08 |

BW, body weight. BT, body temperature. The data are shown as mean ± SEM. There were 9 rats in each group. **p* < 0.05, significant change from initial body weight measured at Inj1. There were no between group differences for any body weight value measured thereafter (*p* > 0.05, for all comparisons).

**Supplementary Table S4**

Body weights and body temperatures at key points of control (i.e., vehicle, no fentanyl) study

| **Parameter** |  | **Treatment groups** | | | | |
| --- | --- | --- | --- | --- | --- | --- |
| **Body Weight, grams** |  | **Vehicle** |  | **D-CYSee** |  | **D-CYSea** |
| Pre |  | 336 ± 1.7 |  | 335 ± 1.2 |  | 335 ± 1.9 |
| Post-Inj 5 |  | 340 ± 1.9 |  | 339 ± 1.2 |  | 340 ± 2.3 |
| Post-Inj 10 |  | 340 ± 2.1 |  | 344 ± 1.3 |  | 346 ± 2.9 |
| Post-NLX |  | 343 ± 2.2 |  | 343 ± 1.2 |  | 345 ± 2.8 |
| Δ1. Post-Inj 5 *vs* Pre |  | +4.4 ± 0.6* |  | +4.6 ± 0.8* |  | +5.7 ± 0.6* |
| Δ1. Post-Inj 10 *vs* Pre |  | +7.8 ± 0.9* |  | +9.6 ± 0.9* |  | +11.1 ± 1.4* |
| Δ3. Post-Inj10 *vs* Post-Inj 5 |  | +3.3 ± 0.6* |  | +5.0 ± 0.6* |  | +5.4 ± 0.8* |
| Δ4. Post-NLX *vs* Post-inj 10 |  | -0.9 ± 0.7 |  | -1.3 ± 0.7 |  | -0.9 ± 0.6 |
| **Body Temperature, ^o^C** |  | **Vehicle** |  | **D-CYSee** |  | **D-CYSea** |
| Pre |  | 37.5 ± 0.06 |  | 37.6 ± 0.08 |  | 37.4 ± 0.06 |
| Post-Inj 5 |  | 37.4 ± 0.06 |  | 37.5 ± 0.06 |  | 37.4 ± 0.05 |
| Post-Inj 10 |  | 37.5 ± 0.07 |  | 37.6 ± 0.04 |  | 37.5 ± 0.07 |
| Post-NLX |  | 37.6 ± 0.12 |  | 37.7 ± 0.10 |  | 37.6 ± 0.07 |
| Δ1. Post-Inj 5 *vs* Pre |  | -0.10 ± 0.08 |  | -0.08 ± 0.10 |  | +0.02 ± 0.08 |
| Δ1. Post-Inj 10 *vs* Pre |  | -0.10 ± 0.10 |  | +0.03 ± 0.08 |  | +0.09 ± 0.08 |
| Δ3. Post-Inj10 *vs* Post-Inj 5 |  | +0.12 ± 0.8 |  | +0.11 ± 0.07 |  | +0.07 ± 0.08 |
| Δ4. Post-NLX *vs* Post-inj 10 |  | +0.03 ± 0.08 |  | +0.07 ± 0.08 |  | +0.09 ± 0.06 |

NLX, naloxone hydrochloride (1.5 mg/kg, IV). MAP, mean arterial blood pressure. D-CYSee, D-cysteine ethyl ester (250 μmol/kg, IV). D-CYSea, D-cysteine ethyl amide (100 μmol/kg, IV). The data are shown as mean ± SEM. There were 9 rats in each group. **p* < 0.05, significant change from Pre-values. Note that there were no between-group differences for any parameter (*p* >0.05, for all comparisons).
